# A novel framework for automated warehouse layout generation

**DOI:** 10.3389/frai.2024.1465186

**Published:** 2024-10-15

**Authors:** Atefeh Shahroudnejad, Payam Mousavi, Oleksii Perepelytsia, Sahir, David Staszak, Matthew E. Taylor, Brent Bawel

**Affiliations:** ^1^Amii (Alberta Machine Intelligence Institute), Advanced Technology Department, Edmonton, AB, Canada; ^2^Routeique Inc, Calgary, AB, Canada; ^3^Department of Computing Science, University of Alberta, Edmonton, AB, Canada

**Keywords:** AI, constrained optimization, automation, warehouse design, logistics

## Abstract

Optimizing warehouse layouts is crucial due to its significant impact on efficiency and productivity. We present an AI-driven framework for automated warehouse layout generation. This framework employs constrained beam search to derive optimal layouts within given spatial parameters, adhering to all functional requirements. The feasibility of the generated layouts is verified based on criteria such as item accessibility, required minimum clearances, and aisle connectivity. A scoring function is then used to evaluate the feasible layouts considering the number of storage locations, access points, and accessibility costs. We demonstrate our method's ability to produce feasible, optimal layouts for a variety of warehouse dimensions and shapes, diverse door placements, and interconnections. This approach, currently being prepared for deployment, will enable human designers to rapidly explore and confirm options, facilitating the selection of the most appropriate layout for their use-case.

## 1 Introduction

The main goal of Warehouse Management Systems (WMS) is running operations as efficiently as possible to improve profitability through increasing productivity, reducing labor costs, and ultimately increasing customer satisfaction. While the majority of the literature aiming to boost WMS efficiency focuses on improving slotting, order sequencing, and fulfillment methods (Boysen and de Koster, 2024), another key component of a WMS is optimal space utilization. It reduces the need for a larger capacity warehouse by maximizing inventory storage and minimizing wasted or underutilized areas. Moreover, warehouse configuration has a direct impact on all warehouse operations, especially the worker routing and picking processes. Efficient warehouse configurations (i.e., layouts) can enhance the order fulfillment process by eliminating unnecessary movement and related errors, resulting in time and cost savings (Mohamud et al., 2023; Richards, 2017). However, the vast majority of warehouses worldwide still continue to rely on manual management or basic automation (Albert et al., 2023). There are a range of traditional and non-traditional manual layout designs that have been proposed over the years to speed up warehouse operations and minimize operating costs (Bortolini et al., 2020; Kocaman et al., 2021; Zhang et al., 2021). Although manually-designed layouts might be feasible for small warehouses with limited options, for larger scales, they are a less efficient use of warehouse designer time and more prone to human error. Hence, an automated process for candidate layout generation would be beneficial to all stakeholders. Moreover, an automated warehouse layout process would allow users to change specifications over time to meet shifting product demand and facilitate the expansion or restructuring of the warehouse while preserving efficiency and productivity.

To automatically generate optimal candidate layouts, we must first establish clear criteria for what constitutes an optimal layout; In this work, we define an optimal layout as one that maximizes both space usage (storage capacity) and the number of accessible storage points (pick faces), while penalizing the number of long-term storage points (locations that are less desirable due to reduced accessibility), all while adhering to the physical and functional constraints of the space. We aim to design layouts that account for different user preferences among the competing priorities of maximizing storage and accessibility. Therefore, there is generally more than one optimal layout, given the multi-objective nature of the problem. Hence, we must solve a constrained optimization problem that maximizes storage capacity and number of pick faces while penalizing long-term storage points and satisfying a set of constraints. It is worth mentioning that this problem differs from general floor planning, which is a partitioning problem with a given list of rooms and their adjacency, size, and position constraints. Thus, the approaches do not quite apply to our problem.

Unlike automatic floor plan generation (Medjdoub and Yannou, 2000; Che et al., 2017; Wu et al., 2018; Hu et al., 2020; Laignel et al., 2021; Morisset de Pérdigo, 2021; Tamarana and Kumari, 2024; Ślusarczyk et al., 2023), automatic warehouse layout generation has not been fully explored in the literature. In warehouse environments, there are additional challenges not included in those analyzes, such as industrial constraints, changing preferences, and real impact on warehousing operational activities. Prior attempts to automate warehouse layout design have focused on using mathematical optimization methods (Yener and Yazgan, 2019). For example, Zhang and Lai (2006) formulate the problem as an Integer Linear Program (ILP) with the combination of path relinking and a Genetic Algorithm (GA). Gu (2005) uses Generalized Benders Decomposition (GBD) to find the optimal solution. Mathematical approaches have some limitations, such as modeling complexity, lack of flexibility in case of any changes in the requirements, and large computational costs. In recent years, Storage Compact Systems (CCS) have been proposed for more compact warehouses to maximize space utilization by extending in height instead of the surface (Yener and Yazgan, 2023; Tutam et al., 2023; Trost and Eder, 2024). However, the main structure of compact warehouses is different as they use storage towers with no aisles.

We propose a new framework to address the gap in the existing literature and to build a tool applicable to real-world scenarios. We present an interactive and iterative tool that allows warehouse designers to impose operational constraints or preferences and evaluate the optimality using objective measures such as capacity and accessibility. This leads the users to an informed decision on the final configuration based on the existing demands and solves a constrained optimization problem that maximizes storage capacity with fewer long-term storage points and maximizes the number of pick faces while satisfying given warehouse constraints.

## 2 Methodology

**Figure 1:**
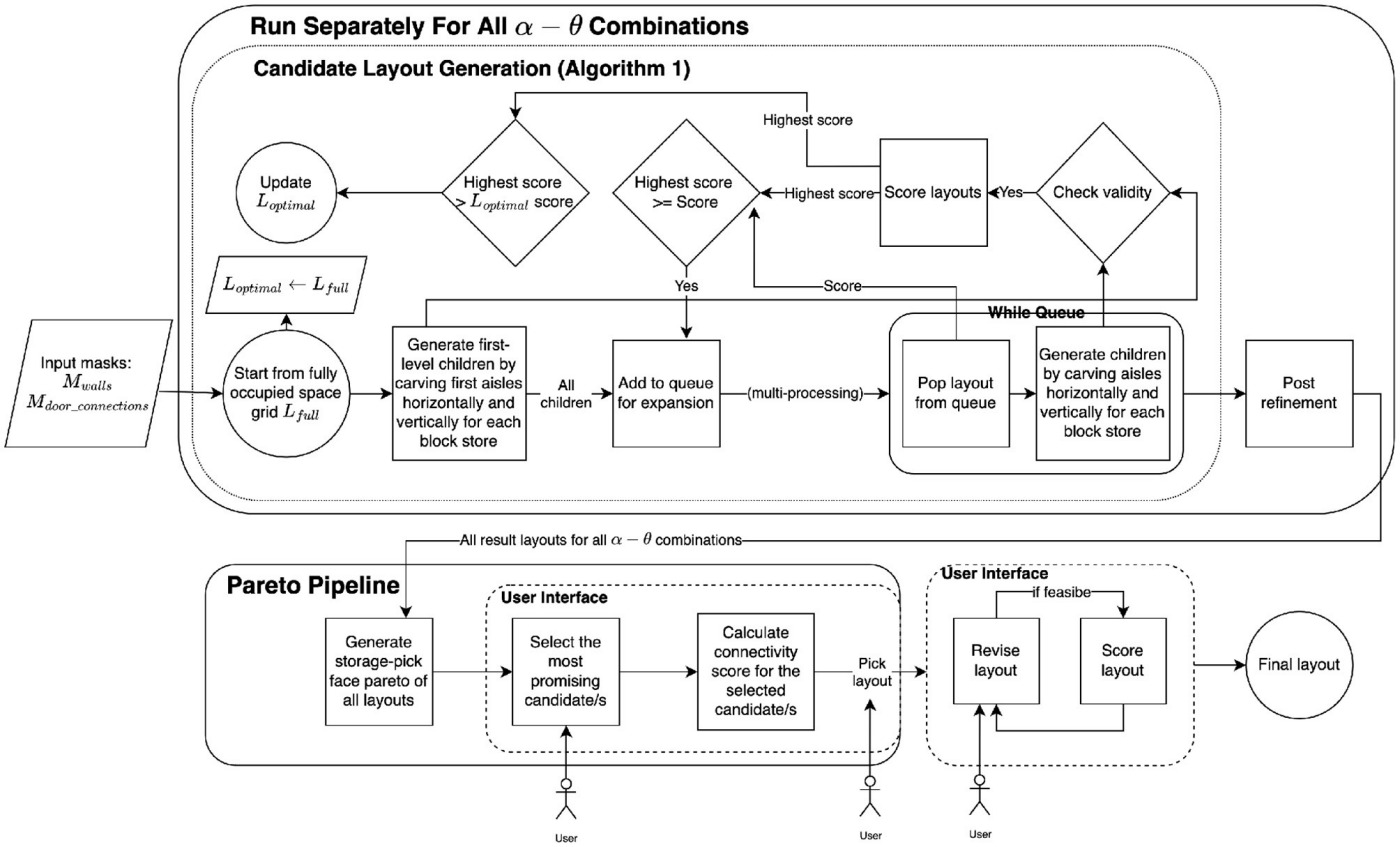
Overview of automated warehouse layout generation framework. The flowchart details the steps from starting with an empty warehouse (with given input masks) through to producing an interactive Pareto plot for warehouse operators to engage with and ultimately select a final layout. Algorithm 1, discussed in more detail in the text, is utilized to generate candidate layouts and is embedded within an iterative process that stores multiple solutions for a range of parameter combinations.

A warehouse consists of multiple spaces (i.e., rooms) that need to be carefully configured for different usage purposes, such as storage or picking. We aim to create space layouts that balance factors such as storage capacity and number of access points tailored to the specific requirements of each space. This would result in easier navigation and better average projected throughput during item retrieval. After generating a range of candidate layouts for a specific space, an experienced warehouse designer can select from the candidates or further refine them. For any candidate chosen in this interactive selection process, the layout would then undergo a thorough validation by an on-site team prior to implementation. The overview of our proposed framework is illustrated in Figure 1. The process is repeated for all warehouse spaces.

**Figure 2:**
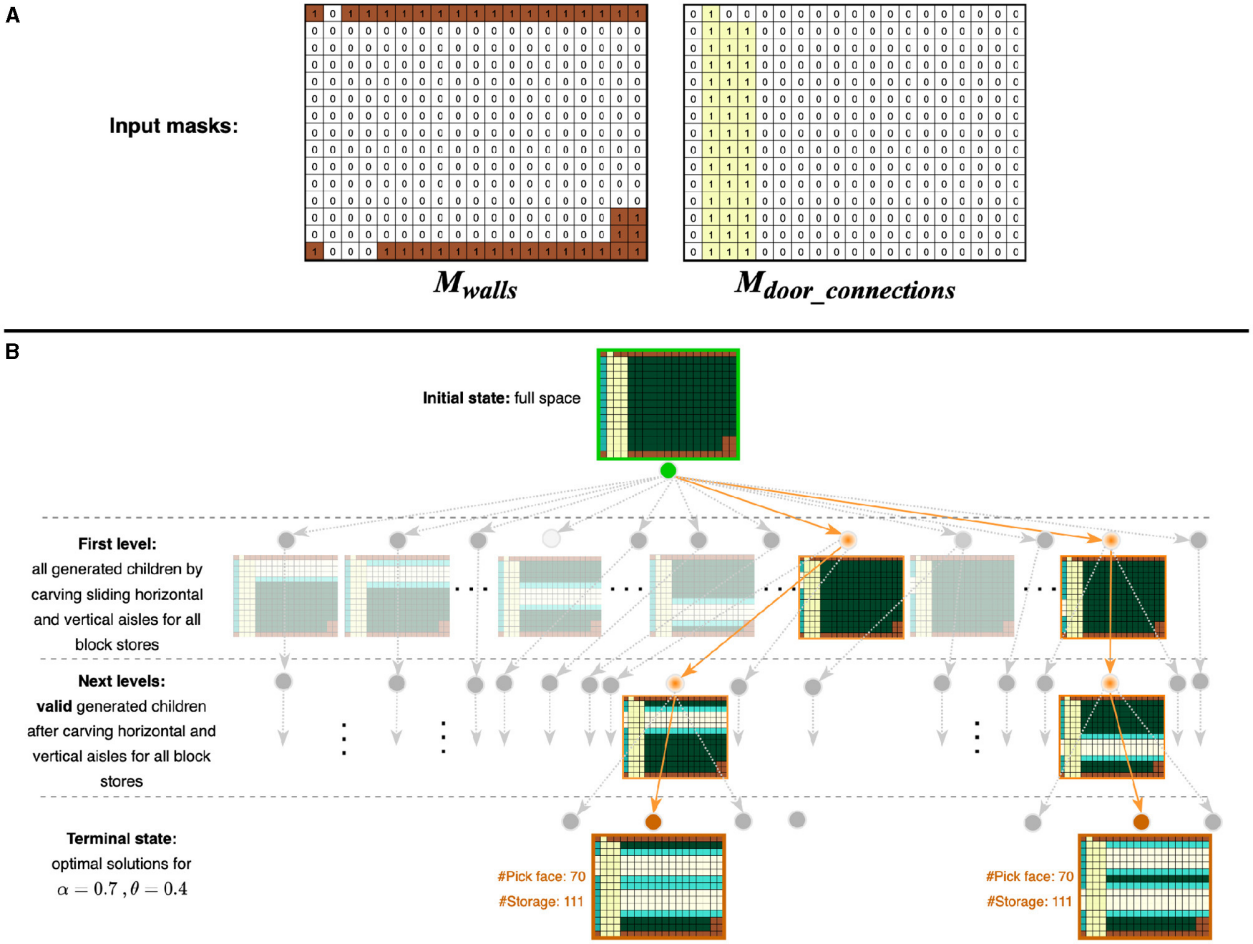
Running example of beam search (*b* = 1) for a sample space with specified aisle width = 3. **(A)** Input masks indicate the positions of walls and door connections respectively. **(B)** The tree search for a sample α = 0.7, θ = 0.4. In the initial state, all available space is designated for storage. At the first level of the search tree, all unique child nodes are generated by carving aisles. This is done by sliding horizontal and vertical aisle templates across all block stores. Depending on the particular α−θ combination specified, different configuration/s may be found as the highest score solution/s for that setting. Here, two children have the same score. The carving process is undertaken again on the chosen configuration/s in subsequent levels until the terminal state is reached. In the figure, colored paths represent routes that lead to the best solutions with the same number of pick faces and number of storage. Space specifications: ■ Walls, ■ Door connections, ■ Aisles, ■ Storage, ■ Pick face.

**Figure 3:**
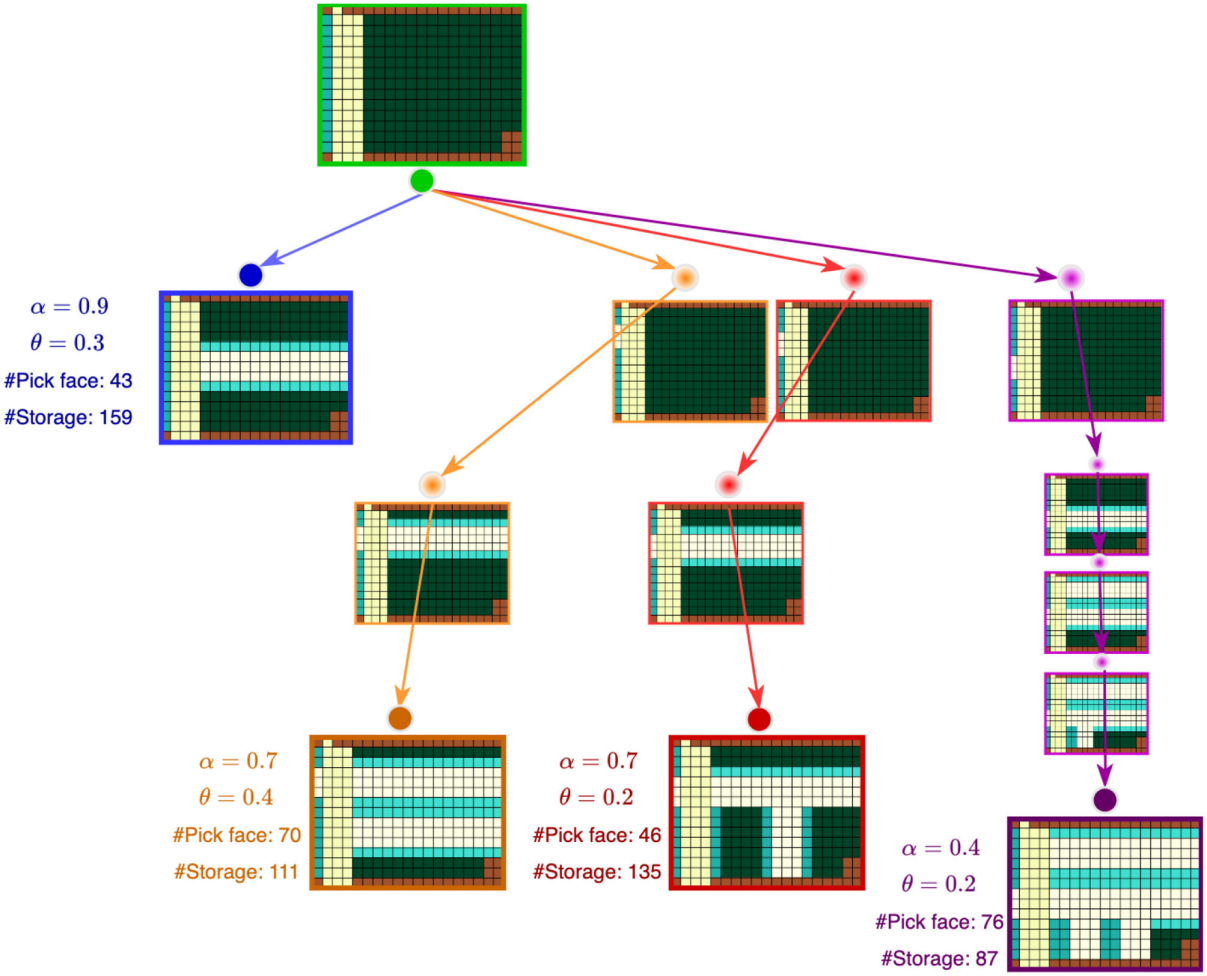
Combined tree searches for different α−θ combinations. Each colored path represents a route that leads to the best solution for a particular α−θ combination. For each of these solutions, the α, θ, number of pick faces, and number of storage are shown. Space specifications: ■ Walls, ■ Door connections, ■ Aisles, ■ Storage, ■ Pick face.

We propose a novel candidate layout generation algorithm (see Algorithm 1) to generate optimal layouts based on tree search. A given space is specified by a discrete two-dimensional grid of cells with two masks marking the positions of walls *M*_*walls*_ and door connections *M*_*door*_*connections*_. Figure 2 shows a running example of a sample space from our industry partner. Each unit cell in the grid is colored based on what category it belongs to: walls, door connections, aisles, storage, or pick face. In the tree search, the grid is initialized with all available cells designated for storage *L*_full_. The tree search then explores the space of possible layouts by systematically carving new aisles (explained in Section 1). Invalid nodes (i.e., layouts violating any constraints) are filtered in the Layout Filtering step (explained in Section 2.2). The valid layouts are scored using a custom scoring function (explained in Section 2.3), and those with the highest score are designated as optimal. As discussed before, there are typically multiple optimal (and viable) layouts for consideration by a customer. In Figure 3, each colored path represents a route that leads to the best solution for a particular setting.

**Algorithm 1:**
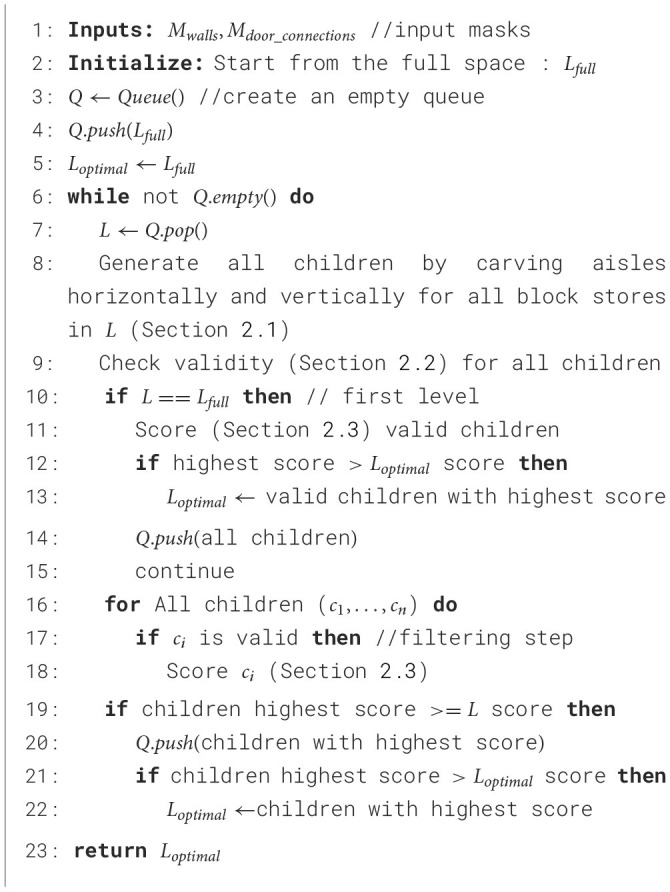
Candidate layout generation (beam size =1).

### 2.1 Tree search

Due to the time-intensive nature and memory constraints of exhaustive tree search at scale, we employ beam search for exploring the tree. Beam search is a heuristic Breadth-First Search (BFS) algorithm that helps to make local decisions and limit the search space. Although beam search yields local optima, it offers a practical and efficient solution considering the constraints and nature of our problem. In the default setting (beam size: *b* = 1), for each node in the tree: (i) all children are generated by carving new potential horizontal or vertical aisles in each block store^1^; (ii) child nodes at the first level are always expanded (to promote diversity of solutions). For deeper levels, only valid child nodes are expanded; and (iii) all valid children are scored and the most promising child (the one with the highest score) is selected for further expansion. The remaining child nodes are pruned. This process continues until there are no more child nodes left for expansion in the tree. This indicates that the terminal state has been reached and the layouts with the highest score are taken to be optimal. For larger beam sizes (*b*>1) however, *b* top-scored children are selected at each level.

To generate all children for a layout node in the tree, we add new potential aisles^2^ by sliding a box (which represents a new aisle) both horizontally and vertically across each block store, freeing up all the cells inside the box. We refer to this process as “carving” because we are removing a portion of the block store and dividing it into two.

### 2.2 Layout filtering

To ensure that only viable and efficient layouts are selected, we sift through all generated children layouts and reject those that violate any functional or efficiency constraints as defined below:

**Functional constraints:**

1. Aisles that are connected to pick faces can not be narrower than the specified aisle width,
2. All aisles need to be reachable by all doors into the warehouse space,
3. No item is allowed to be placed in doorways or areas marked as “reserved,” and
4. No pillar can block an aisle.

**Efficiency constraints:**

5. Aisles wider than the minimum required size are not allowed as they waste space,
6. Two-sided access block stores should contain at least two rows, and
7. Each block store should contain more than one item as it is never efficient or desirable to store a single item at a given location.

### 2.3 Layout scoring

Candidate solutions are evaluated and the underperforming tree nodes are pruned. The scoring function is a critical component used as a heuristic in the tree search. A misspecified score will be detrimental to the node expansion of the tree search resulting in sub-optimal solutions. We introduce a scoring function (Equation 1), which not only enables trade-offs between important performance factors (e.g., storage and accessibility) but also facilitates diverse layout generation. We use normalization and define the term weights carefully in the scoring function to ensure the layout assessment is accurate and unbiased. Formally, we aim to solve the following optimization problem:


(1) $$\begin{array}{ll} \text{maximize } & Score=\alpha T_{s}+\beta T_{pf}+c_{1}T_{o}\\ \text{subject to } & \text{Constraints }(1)-(7), \end{array}$$

where the constraints have been listed in Section 2.2. The scoring function is a weighted combination of three terms, namely, the normalized storage capacity *T*_*s*_, the normalized number of pick faces *T*_*pf*_, and the normalized number of block stores in a specific orientation (vertical or horizontal) *T*_*o*_. The coefficients α and β specify the relative importance of the first two terms, while the coefficient *c*_1_ is a fixed hyperparameter selected empirically (more details later).

The normalized storage capacity *T*_*s*_ is defined as,


(2) $$\begin{array}{l} T_{s}=\frac{N_{s}-c_{2}\theta P_{a}}{Total\;open\;area}, \end{array}$$

where *N*_*s*_ is the storage capacity; θ is a weighting coefficient; *c*_1_ and *c*_2_ are hyperparameters (discussed in more details later), and *P*_*a*_ is an accessibility penalty defined as,


(3) $$\begin{array}{l} P_{a}:=\underset{BS_{i}}{\sum }\omega_{i}\underset{j\in BS_{i}}{\max}{\left(h_{j}-1\right)}^{2}, \end{array}$$

with ω, and *h* corresponding to the width and item heights of a block store *BS*. The penalty is related to the number of items that need to be removed to access the deepest row in a block store. The chosen quadratic scaling has desirable symmetry properties ensuring that higher depths are appropriately penalized.

The second term in Equation 1, *T*_*pf*_, the normalized number of pick faces is defined as,


(4) $$\begin{array}{l} T_{pf}=N_{pf}/N_{s}, \end{array}$$

where *N*_*pf*_ is the number of pick faces.

Finally, the third term in Equation 1, *T*_*o*_ represents the normalized number of block stores in a specific orientation (vertical or horizontal) if that orientation is opposite to the space orientation. Without using this term, block stores tend to be aligned with the space orientation. However, sometimes the opposite orientation is preferable due to the location of the staging area^3^. *T*_*o*_ provides a control to allow the dominated layout orientations thereby promoting more diversity in the results.

The weighting coefficients, α, β, and θ, adjust the balance between the different terms. The term α∈{0.1, …, 1} controls the balance between storage capacity and number of pick faces, β = min(0.1, 1−α), and θ∈{0.1, …, α/2} are defined based on α. The term θ controls the accessibility penalty *P*_*a*_. The maximum of θ is set based on α to avoid over-penalizing. Hyperparameters *c*_1_ = 0.01 and *c*_2_ = 0.1 are set empirically to scale the corresponding terms.

Each combination of α and θ represents a particular preference for the properties of the generated optimal layout/s discovered by the tree search. The process for selecting the final layout involves the warehouse designer, and is discussed in more details in Section 3.

### 2.4 Connectivity score

In addition to the scoring function presented above, we define another score term, connectivity to estimate the likely relative throughput expected from different candidate layouts. Note that the exact throughput cannot be known *a priori* as it will depend on specific product assignment and order lists. Our estimates here are used only to determine the ranking order of different candidates as a tool to select among several optimal candidates generated above. The connectivity score is defined as the average cumulative ratios of shortest distances to the Manhattan distances (Black, 2006) for pairs of pick faces:


(5) $$\begin{array}{l} C=\frac{1}{N_{pf}}\underset{i\leq j}{\sum }\frac{1}{D_{i,j}^{Shortest}/D_{i,j}^{Manhattan}}, \end{array}$$

where *N*_*pf*_ is the number of pick faces, and *D*_*i, j*_ is the distance (i.e., shortest or Manhattan) between two pick face locations.

This is based on the intuition that for a high-throughput (i.e., more connected) layout, the shortest distances (between pairs of pick faces) will on average be closer in value to the Manhattan distance. This function has the additional desired property that it is normalized to one, facilitating simple direct comparisons between different layouts. Note that we decided not to include connectivity as an independent scoring term in Equation 1 to simplify the process and minimize instabilities in the search.

### 2.5 Post-refinement

The objective of the post-refinement step is to apply any additional constraints to arrive at the final layouts. While every application will require some customization by the on-site team often these constraints and requirements can be codified to save time. In one application, the pallet racking system only allowed even numbers of racking units along the total block store (due to how the racking infrastructure was constructed). In another application, clear paths were necessary to access pillars that contained fire safety equipment. In both cases, the flexibility of our algorithm allowed for these constraints to be programmatically applied as a final step, only passing layouts that fulfilled the criteria.

### 2.6 Implementation details

The computational complexity of our method primarily depends on the beam size, space dimensions, and aisle width. We use Beam Search, a variant of Breadth-First Search (BFS), which explores all children at each depth level and selects a beam of highest-scoring valid children. The time and space complexity are both O(bd), where *b* is the beam size (number of candidates kept at each step), *d* = max(1, ⌊*h*/(aisle width+1)⌋+⌊*w*/(aisle width+1)⌋) is the maximum tree depth, and *h* and *w* are the height and width of the space. The space complexity arises from the need to store the best *b* candidates at each of the *d* levels of the search tree.

By increasing the beam size, the search space is expanded as more children are explored at a time. However, it also increases the exploration time. In practice, *b* = 1–3 showed a good balance between performance and computational efficiency depending on the space size.

Moreover, we use multiprocessing to expedite the search process, especially for larger beam sizes. We used eight CPU cores with 16 GB of memory to run experiments. The average processing time for generating the Pareto plot for a small-sized space (16 × 23) is 33 s, for a medium-sized space (28 × 21) is 82 s, and for a large-sized space (17 × 47) is 954 s.

## 3 Results

**Figure 4:**
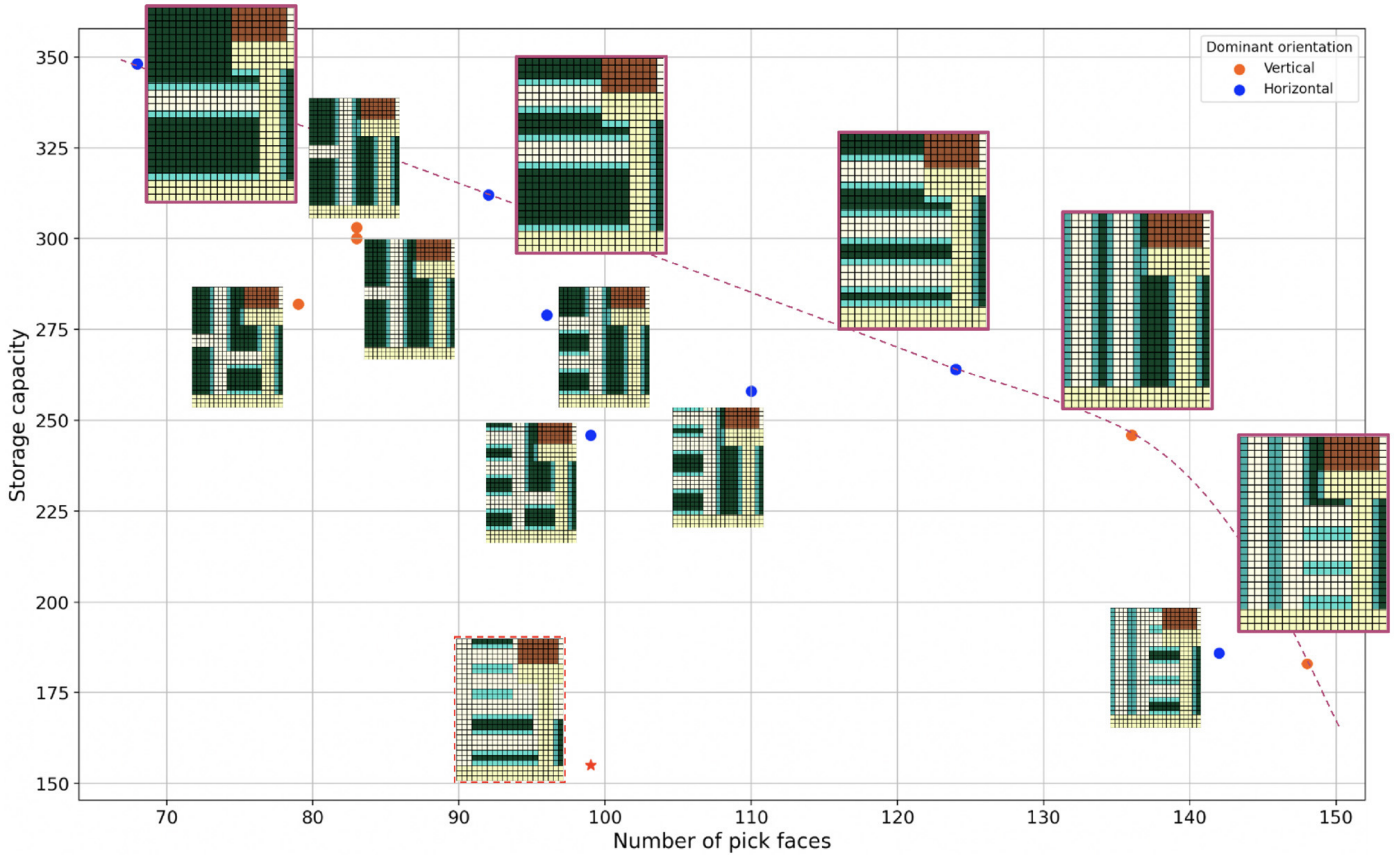
Pareto visualization for a medium-sized space from our industry partner with specified door connections and aisle width = 3. The pink dashed line shows the Pareto front. Zoomed layouts correspond to the data points on the Pareto front. The red star at the bottom shows the manually-designed layout which has been specified by the red dashed border. Space specifications include the following: ■ Walls, ■ Door connections, ■ Aisles, ■ Storage, ■ Pick face.

To generate all possible optimal layouts for a given space, the layout generation process is run separately for all combinations of α and θ in their defined ranges. Drawing from the pool of generated optimal layouts, we create a Pareto plot that visualizes possibilities with respect to the storage capacity and number of pick faces. The Pareto plot is used as an interactive decision-making tool. Figure 4 shows the Pareto plot for a medium-sized space from our industry partner. The Pareto front comprises optimal layouts that dominate the other candidates by striking a better trade-off between the storage capacity and number of pick faces. The Pareto front is downward sloping, illustrating that the storage capacity decreases with increasing number of pick faces. When two candidate layouts have the same score in the same α−θ setting (e.g., Figure 2B), the connectivity score comes into play and decides which one is likely to lead to a higher-throughput design.

In Figure 4, we compare the auto-generated optimal layouts in the Pareto plot with the existing manually-designed layout (indicated by the red star added to the plot). We observe notable improvements across both number of pick faces and storage capacity.

We closely collaborated with expert warehouse designers to validate the quality of the generated layouts and compare the results with manually-generated versions. The designers reported that the process was user-friendly and served as an effective collaborative tool, significantly streamlining their efforts to achieve good layouts.

## 4 Discussion

Warehouse layout design plays a vital role in warehouse operations performance. We proposed a novel automated optimal layout candidate generation framework using beam search that satisfies a set of constraints. We also introduced a new scoring function that handles a balance between storage capacity, number of access points, and accessibility cost. Our method can generate a wide variety of candidate layouts for different ranges of picking and storage areas and we demonstrated this in various spaces of two real-world warehouses. The simplicity of the method makes it easily adaptable to any changes in user specifications and requirements.

Despite all these strengths, our approach is not without limitations. One limitation is that we were unable to measure the throughput of the layouts and compare their performances comprehensively. Throughput depends on order lists and item allocation, which would add a layer of complexity that is beyond the scope of this work. As throughput is the ultimate measure of effectiveness, not being able to account for these factors restricts our ability to fully evaluate the efficiency of the different layouts. Despite this limitation, the proposed solution was tested on nine available physical spaces through the partnership between Amii and Routeique. In the experiments, the framework generalized well to both large and small spaces as well as those with non-rectangular shapes and the expert warehouse designers verified the feasibility and quality of the generated layouts.

Finally, we did not incorporate some relevant constraints, for example varying heights of rows, and larger-sized doorways/entryways. However, we found that it was relatively straightforward to programmatically add those additional constraints for the unique artifacts found in spaces such as fire extinguishers (see Section 2.5). While it is challenging to comprehensively anticipate and account for all unique elements found in specific real-world scenarios, our method is flexible and could incorporate the majority of such constraints.

In the future, we will continue to validate and refine our tool in more diverse settings and in different warehouses. Feedback from a larger number of warehouse designers will guide the direction of future development.

## Data Availability

The datasets presented in this article are not readily available because, they represent the physical specifications from real-world warehouses and are an internal Routeique dataset. Requests to access the datasets should be directed to: atefeh.shahroudnejad@amii.ca.

## Footnotes

^1^A group of contiguous storage cells is called a block store.

^2^Aisles are pathways that connect block stores to door connections and do not contain any stored items (all cells within aisles are empty). They have a fixed width but can vary in length.

^3^Staging area is where items are loaded to or unloaded from the warehouse.
